# Cardiovascular Findings in Klippel-Feil Syndrome: A Systematic Review

**DOI:** 10.7759/cureus.72540

**Published:** 2024-10-28

**Authors:** Abbigail Niewchas, Salma Alkhatib, Christopher Stewart, Mitchell Fisher, Randall Hansen, Alex L Otto, Kent McIntire, Suporn Sukpraprut-Braaten

**Affiliations:** 1 Medical School, Kansas City University of Medicine and Biosciences, Joplin, USA; 2 Otolaryngology, Head and Neck Surgery, Freeman Health System, Joplin, USA; 3 Graduate Medical Education, Unity Health, Searcy, USA; 4 Graduate Medical Education, Kansas City University, Kansas City, USA

**Keywords:** cardiac, cardiovascular, congenital heart defect, kfs, klippel-feil syndrome

## Abstract

Klippel-Feil syndrome (KFS) is a congenital disease defined by an abnormal fusion between cervical vertebrae. Due to the rarity of the disorder, its prevalence, along with its pathogenesis and associated conditions, remains to be clearly defined. The aim of this review is to summarize the findings of all case reports of KFS in PubMed over the last 10 years that describe cardiovascular disease, defects, or abnormalities. A total of 43 articles containing 46 reports were included from the 157 case reports considered. Cases were reviewed for commonality in biological sex and vertebral fusion and level using the Samartzis classification system to determine what association, if any, exists with the cardiovascular findings analyzed. A total of 72% of cases reported one or more findings consistent with congenital heart disease. Using the Samartzis classification system, type III KFS was the most common fusion profile overall in this subset of patients. The heterogeneity of disease manifestations makes the treatment and management of KFS case-dependent, though current guidelines highlight the importance of a multidisciplinary care team for pediatric patients. Our findings support this notion and provide evidence for the inclusion of a care provider who specializes in cardiovascular medicine in patients of all ages, as well as the consideration of additional diagnostic screening exams for cardiovascular abnormalities. Future studies into the embryological origin of KFS and a more robust search for a genetic marker are needed to better understand the development of the disease and its various associated conditions.

## Introduction and background

Klippel-Feil syndrome (KFS) is a rare congenital disease characterized by an abnormal fusion of two or more cervical vertebrae [1]. First described in 1912 by Dr. Maurice Klippel and Andre Feil, it was defined by the common triad of a short, webbed neck, limited range of motion in the cervical spine, and low posterior hairline [2]. Since its designation, it has been found that less than half of patients diagnosed with KFS present with the common triad [3]. Consequently, there have been efforts by others in the field to re-establish a classification system that better encompasses the heterogeneity of clinical presentations. The Samartzis classification system divides KFS into three types based on the number and level of vertebral fusion(s) and can be used by physicians to estimate the severity and presentation of symptoms in this subset of patients [4]. 

In addition to the variable phenotypic presentation seen in KFS, further research into aberrations in other organ systems has similar findings [5]. Among these are cardiovascular abnormalities with varying severity and forms. Though dozens of case reports, like those analyzed in this review, have highlighted cardiovascular findings in Klippel-Feil patients, there is no review of the literature summarizing frequent cardiovascular abnormalities, defects, or diseases seen in concordance with the syndrome. Additionally, there has been no discussion of whether the Samartzis classification or biological sex of the patient has any correlation with the cardiovascular findings. The aim of this review is to describe the most common cardiovascular findings in patients with KFS to establish a baseline knowledge of this association and to provide reference material for clinicians managing KFS.

## Review

Methods

Information Sources and Strategy 

This systematic review was prepared according to the protocol set forth by the Preferred Reporting Items for Systematic Reviews and Meta-analyses (PRISMA) guidelines [6]. In September of 2023, an electronic search was conducted on the PubMed database to identify case reports published in the last 10 years containing the keyword “Klippel-Feil Syndrome” that also described a cardiovascular abnormality, defect, or disease.

Eligibility Criteria 

To classify the cardiovascular findings analyzed in this review, the American Board of Internal Medicine’s (ABIM) Cardiovascular Disease Examination Blueprint was utilized [7]. A “cardiovascular finding” was identified if the case report contained a specific description that fell into one of the nine overarching categories described by the ABIM Blueprint, including arrhythmias, coronary artery disease, heart failure or cardiomyopathy, valvular disease, pericardial disease, congenital heart defect, vascular disease, systemic hypertension or hypotension, pulmonary circulation disorder, or a systemic disorder affecting the circulatory system. An additional category deemed “other” was created to include the articles that mentioned a cardiovascular finding but did not provide adequate information for it to be categorized. 

Study Selection

Full-text screening of the case reports identified with the initial search criteria, described in the Information Sources and Strategy section, was performed independently by two reviewers (AN and SA). Those that had no description of a cardiovascular finding or a description of a cardiovascular finding in a genus other than human were excluded (two studies were reported in canines). Disagreements were resolved by consensus.

Data Analysis

To generate a clinically applicable set of data, cases were analyzed by several additional variables to determine what association, if any, exists with the categories of cardiovascular disease used in this review. First, cases were contrasted based on the biological sex of the patient, where “female” is used to describe patients assigned female at birth (AFAB) and “male” is used to describe patients assigned male at birth (AMAB). Cases that did not specify the sex of the patient were classified as “unknown” when discussing biological sex. To investigate the possibility of an association between the number and level of cervical fusions with the cardiovascular findings identified, cases were further classified based on vertebral fusion profile using the fusion levels themselves as categories as well as using the Samartzis classification system [4]. The Samartzis classification system categorizes Klippel-Feil cases into types I, II, and III based on the number and level of cervical fusions. Cases were also analyzed by age to investigate a possible association with the cardiovascular findings. The age range of cases analyzed was from 22 weeks’ gestation to 70 years old, which were ultimately divided into three categories; “youngest” which includes cases where the patients' age ranged from 22 weeks’ gestation to 36 months old, “middle” which included cases where the patients' age ranged from 36 months old to 18 years old, and “oldest” which included cases where the patients' age ranged 18 years old to 70 years old. Finally, cases were reviewed for genetic testing results to determine if any association exists with the cardiovascular diseases analyzed.

Quality Assessment and Risk of Bias

To uphold the quality of this systematic review and prevent potential bias from its design and analysis, an appraisal process has been incorporated using the Joanna Briggs Institute (JBI) critical appraisal checklist for systematic reviews and research syntheses [8]. Two reviewers worked independently to discuss and assess the criteria and ensured eligibility of cases based on quality. Firstly, explicit statement of the purpose and intention of the review was noted along with clear and detailed outlining of the inclusion criteria. Limitations placed on the search for articles included only studies describing cases in human patients and published within the date range of October 2013-January 2023. A potential risk in the quality assessment is the risk of bias due to the use of only one electronic database (PubMed) as this may have led to an incomprehensive search strategy. Recommendations made for cardiology referrals and imaging were verified to be supported by the original reported data, and direction for future research of cardiovascular presentations in KFS was made clear. 

Results

Study Selection

**Figure 1:**
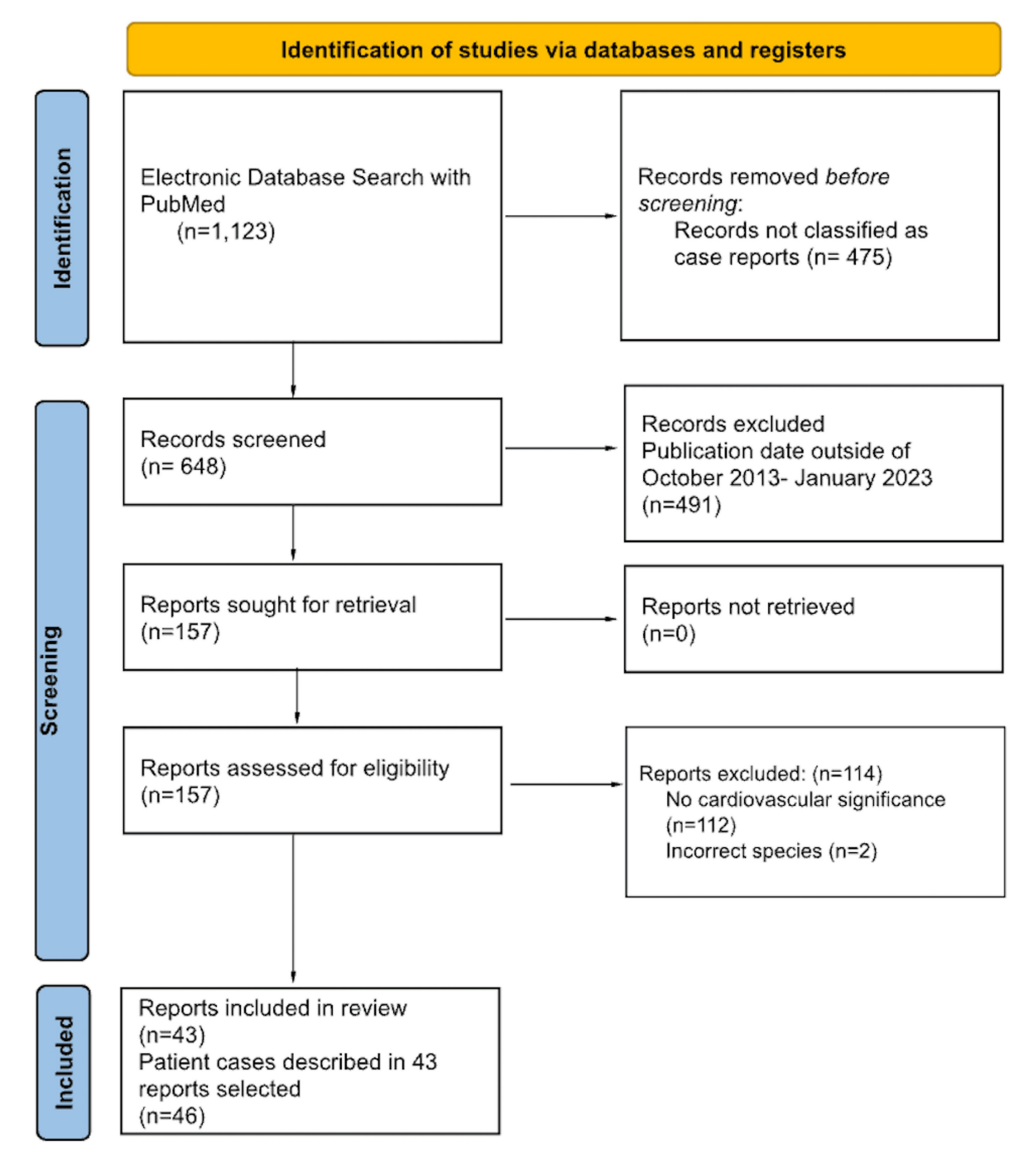
PRISMA flow chart for article selection process PRISMA: Preferred Reporting Items for Systematic Reviews and Meta-analyses

Figure 1 shows a flow diagram of the article selection process. The initial search was conducted in PubMed using the keyword “Klippel-Feil Syndrome.'' This generated 1,123 articles, of which 648 were identified as case reports using the “article type” filter. All publications that did not identify as case reports in this manner were excluded. Of the 648 case reports, 157 were published between October 2013 and January 2023. Using the eligibility criteria outlined in the Eligibility Criteria section, 43 articles (46 patients) were selected for final inclusion [9-51]. The discrepancy in final report number and patient case number is attributable to the fact that one report described four patient cases. 

Specific Cardiovascular Diagnoses

The Eligibility Criteria section outlines the general categorization of cardiovascular diseases analyzed. Within each category, several subcategories with specific cardiovascular diagnoses are described. Subcategories under the "arrhythmias" category includes diseases resulting in abnormal atrioventricular conduction, sinus node dysfunction, supraventricular arrhythmias, ventricular arrhythmias, atrial arrhythmias, and channelopathies, among others. The "coronary artery disease" category includes angina pectoris, chronic ischemic heart disease, acute myocardial infarction, early complications following acute myocardial infarction, atypical angina, or noncardiac chest pain. The "heart failure and cardiomyopathy" category includes heart failure, cardiomyopathies, heart transplantation, and mechanical circulatory support. The "valvular disease" category describes any disorder involving the aortic, pulmonary, tricuspid or mitral valve, including endocarditis, cardiac murmurs, and other cardiac sounds. The "pericardial disease" category includes acute pericarditis, chronic pericarditis, pericardial constriction, or pericardial effusion. The "congenital heart disease" category includes congenital malformations of the cardiac chambers and connections, the cardiac septa, the aortic, pulmonary, tricuspid or mitral valves, and the great arteries or veins, among others. The "vascular diseases" category includes cerebrovascular diseases; diseases of the arteries, arterioles, and capillaries; and diseases of the veins, lymphatic vessels, and lymph nodes. The "systemic hypertension and hypotension" category includes hypertensive or hypotensive diseases. The "pulmonary circulation disorders" category includes pulmonary hypertension or pulmonary embolism. Finally, "systemic disorders affecting the circulatory system" is a broad category that includes a variety of diseases affecting a diverse set of body systems, benign and malignant neoplasms, injuries and poisoning, and secondary cardiovascular conditions that develop as a result. A complete list of the specific diagnoses of cardiovascular disease included within each subcategory is publicly available in the online version of the ABIM Cardiovascular Disease Exam Blueprint [7].

The Samartzis Classification System 

The Samartzis classification system of KFS differentiates cases based on the number and level of cervical fusion(s) and used group cases of a similar fusion profile. According to this system, type I KFS is defined as having a single congenitally fused cervical segment; type II KFS is defined as multiple noncontiguous, congenitally fused segments; and type III KFS is defined as multiple contiguous, congenitally fused cervical segments [4]. 

Cardiovascular Findings

**Figure 2:**
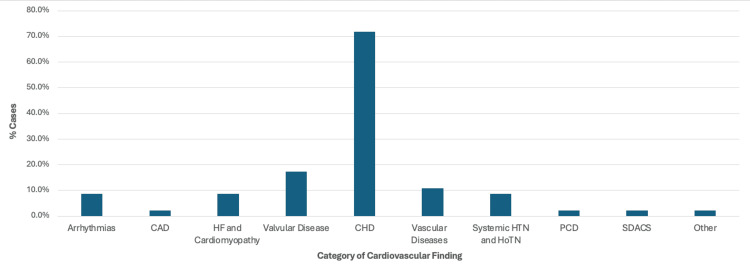
Percentage of case reports containing one or more cardiovascular finding(s) described in each category of the American Board of Internal Medicine's Cardiovascular Disease Examination Blueprint, or in the "other" category CAD: Coronary artery disease; HF: heart failure; CHD: congenital heart disease; HTN: hypertension; HoTN: hypotension; PCD: pulmonary circulation disorders; SDACS: systemic disorders affecting the circulatory system

A case-by-case summary of the cardiovascular findings within each category of cardiovascular diseases can be found in Table 1, in the order of patient age from the youngest to the oldest. The specific diagnosis associated with each case can be found in the supplemental data. In addition, Figure 2 provides the frequency of each category of cardiovascular diseases analyzed. The next subsections (Arrhythmias- Systemic Disorders Affecting the Circulatory System) will analyze the results based on the nine categories set forth by the ABIM Cardiovascular Examination Blueprint, focusing on the distribution of cases based on biological sex and the Samartzis classification as well as the most common findings in each category. 

**Table 1:** Summary of cardiovascular findings in the 46 cases analyzed A (+) indicates one or more finding in the specified category of cardiovascular disease

	Age and sex	Vertebrae fusion and level	Samartzis classification	Arrhythmias	Coronary artery disease	Heart failure and cardiomyopathy	Valvular disease	Pericardial disease	Congenital heart disease	Vascular diseases	Systemic hypertension and hypotension	Pulmonary circulation disorders	Systemic disorders affecting the circulatory system	Other
Bisht et al. [9]	22 weeks’ gestation F	Small and fused vertebral bodies at cervical and upper thoracic spine	Type II						(+)					
Pirino et al. [10]	22 weeks’ gestation F	C1-C5	Type III						(+)					
Alaqeel et al. [11]	Newborn F	C5-C7	Type III						(+)					
Hernando et al. [12]	Newborn M	Not specified	Unknown						(+)					
Bejiqi et al. (1) [13]	2-day old M	C2-C3	Type I			(+)								
Bejiqi et al. (2) [13]	2-day old M	Massive cervical fusion	Type III						(+)					
Altay et al. [14]	26-day old F	Not specified	Unknown						(+)			(+)		
Hitosugi et al. [15]	1 year old F	C2-C3	Type I						(+)					
Brunet et al. [16]	16-month-old M	Not specified	Unknown						(+)					
Bejiqi et al. (3) [13]	28-month-old F	C2-C3	Type I	(+)					(+)					
Kandemirli et al. [17]	8-year-old F	C2-C3	Type I						(+)					
Zhang et al. [18]	8-year-old M	AO fusion	Type I	(+)					(+)					
Martirosyan et al. [19]	9-year-old M	C2-C3	Type I						(+)					
Jena et al. [20]	10-year-old F	C5-C7	Type III			(+)	(+)		(+)					
Jasper et al. [21]	11-year-old F	Multiple cervical	Type III						(+)					
Bejiqi et al. (4) [13]	12-year-old F	C1-C2 and C5-C7	Type III	(+)					(+)					
Fontecha et al. [22]	12-year-old F	C1-C5, C6-C7, T1-T2	Type II						(+)					
Isidor et al. [23]	12-year-old F	C2-C3 and C4-C5	Type III						(+)					
Satis et al. [24]	12-year-old M	C1-C7	Type III						(+)					
Abukabbos et al. [25]	13-year-old F	Unknown	Unknown											(+)
Karaca et al. [26]	13-year-old M	C2-C7	Type III						(+)					
Chima-Galan et al. [27]	14-year-old F	C4-C5	Type I				(+)							
Martínez-Quintana et al. [28]	14-year-old M	C2-C4, C7-T1, T1-T2, T2-T3	Type II						(+)					
Ulusoy et al. [29]	15-year-old F	C2-C4	Type III						(+)					
Sidra Naz et al. [30]	15-year-old F	Not specified	Unknown				(+)							
Mohammad et al. [31]	15-year-old M	Multiple cervical segmentation abnormalities	Type III						(+)					
Urdaneta Carruyo et al. [32]	16-year-old M	Partial C4-C5 full C5-C6	Type III						(+)					
Bayram et al. [33]	21-year-old M	C5-C6	Type I						(+)	(+)				
Roberti et al. [34]	22-year-old F	AO, C2-C3, C4-C5, L4-L5	Type II						(+)					
Futane et al. [35]	26-year-old M	AO assimilation, C2-C3	Type II						(+)					
Dialameh et al. [36]	27-year-old U	C3-C5	Type III			(+)	(+)		(+)					
Han et al. [37]	38-year-old F	C2-C3 and C4-C5	Type III							(+)				
Hazra et al. [38]	42-year-old M	C2-C3	Type I						(+)					
Cota et al. [39]	44-year-old M	C2-C6	Type III	(+)			(+)							
Ruzic-Barsic et al. [40]	44-year-old F	C3-C4 and C7-T1	Type II						(+)					
Sato et al. [41]	44-year-old M	C4-C6 partial, T1-T2	Type II							(+)				
Dornbos et al. [42]	45-year-old F	AO assimilation, C2-C3	Type II							(+)				
Ahluwalia et al. [43]	51-year-old M	C1-C3	Type III				(+)							
Sabol et al. [44]	51-year-old F	Not specified	Unknown				(+)		(+)	(+)				
Constantine et al. [45]	52-year-old F	Not specified	Unknown						(+)					
Martinez et al. [46]	52-year-old F	C6-C7	Type I								(+)			
Goebel et al. [47]	53-year-old M	Not specified	Unknown						(+)					
Hammond et al. [48]	54-year-old F	C2-C6	Type III			(+)	(+)		(+)					
Verla et al. [49]	56-year-old F	AO assimilation, dens to T3	Type II								(+)			
Jamrozik et al. [50]	68-year-old M	C2-C3	Type I								(+)		(+)	
Pagano et al. [51]	70-year-old M	Not specified	Unknown		(+)						(+)			

**Figure 3:**
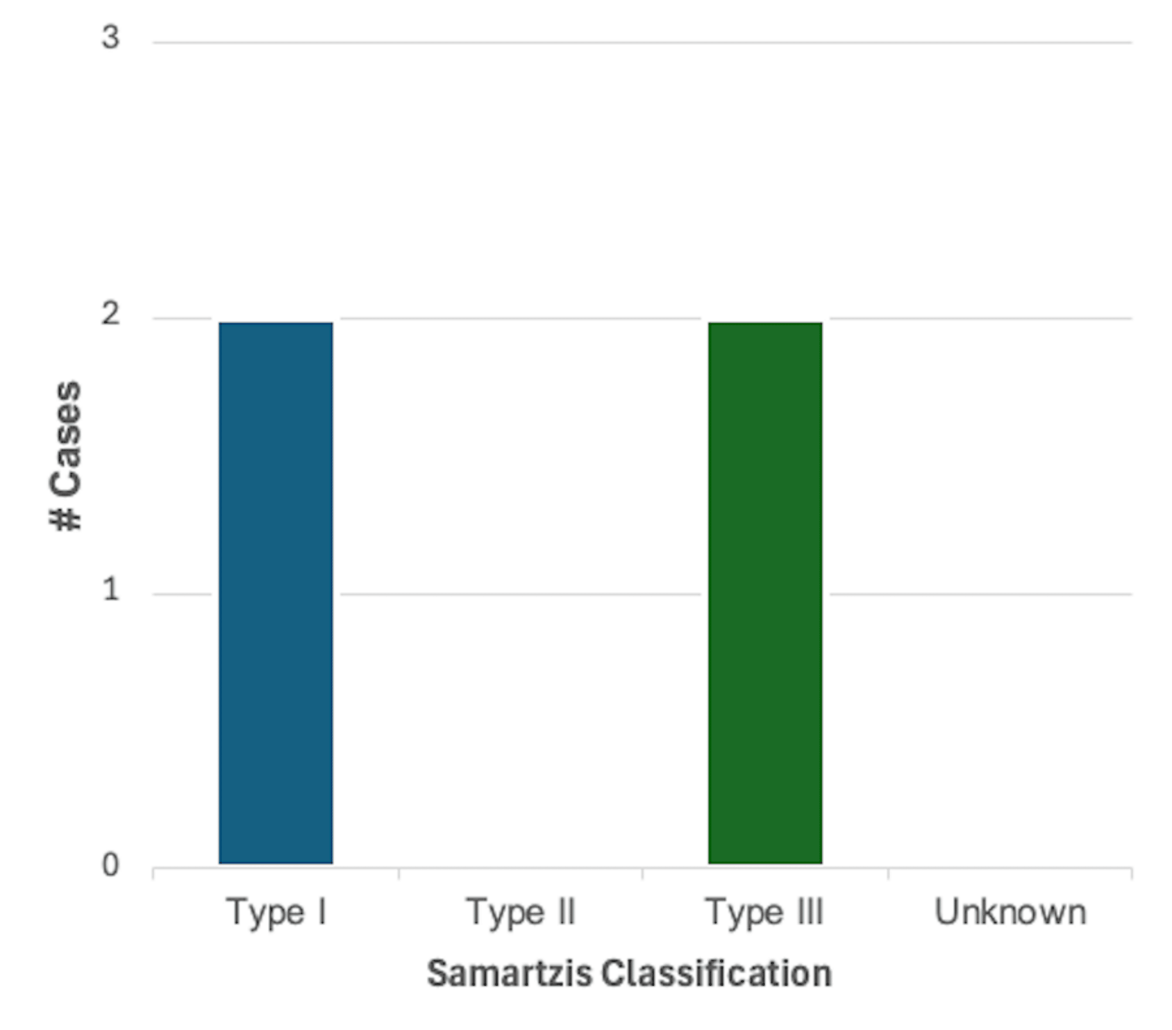
Breakdown of cases reporting one or more findings consistent with arrhythmia (n = 4) based on the Samartzis classification of Klippel-Feil syndrome

Arrhythmias: A description of arrhythmia was reported in 4/46 (8.7%) cases. Of these, 2/4 (50%) were reported in male patients and 2/4 (50%) in female patients. Using the Samartzis classification system, 2/4 (50%) cases were classified as type I KFS, and 2/4 (50%) cases were type III KFS. The most common finding in this category was an incomplete bundle branch block, reported in three cases. A systolic murmur was reported in the other case in this category. A summary of these findings can be found in Figure 3. 

Coronary artery disease: Coronary artery disease was reported in a single case of a male patient with an unspecified vertebral fusion. The patient was 70 years old and had a history significant for placement of a coronary artery bypass graft. 

**Figure 4:**
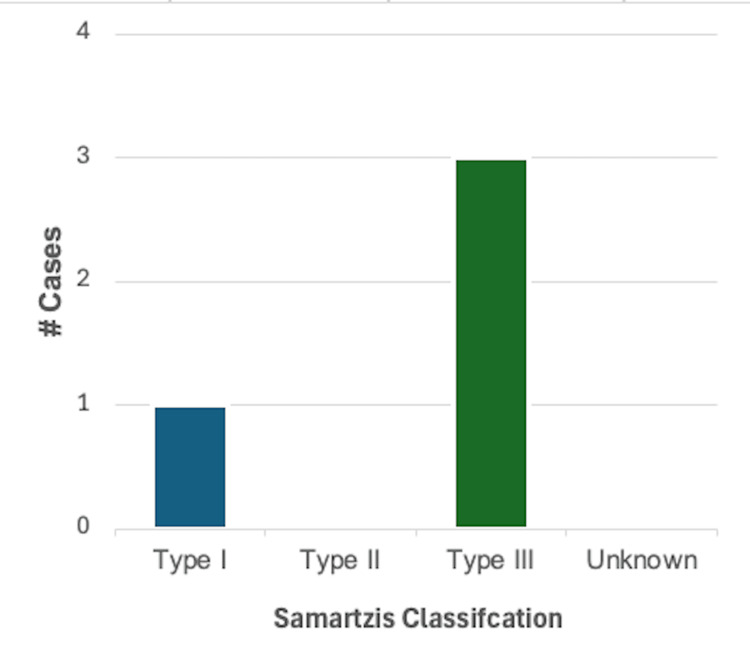
Breakdown of cases reporting one or more findings consistent with heart failure or cardiomyopathy (n = 4) based on the Samartzis classification of Klippel-Feil syndrome

Heart failure and cardiomyopathy: Heart failure or cardiomyopathy was reported in 4/46 (8.7%) cases. Of these, 1/4 (25%) were reported in male patients, 2/4 (50%) in female patients, and 1/4 (25%) cases did not specify the biological sex of the patient. Using the Samartzis classification system, 1/4 (25%) cases were classified as type I KFS, and 3/4 (75%) cases were type III KFS. Left ventricular hypertrophy was specified in two cases, right atrial and ventricular hypertrophy with general cardiomegaly was reported in one case, and an enlarged cardiac silhouette suggesting cardiomegaly was described in another. A summary of these findings can be found in Figure 4. 

**Figure 5:**
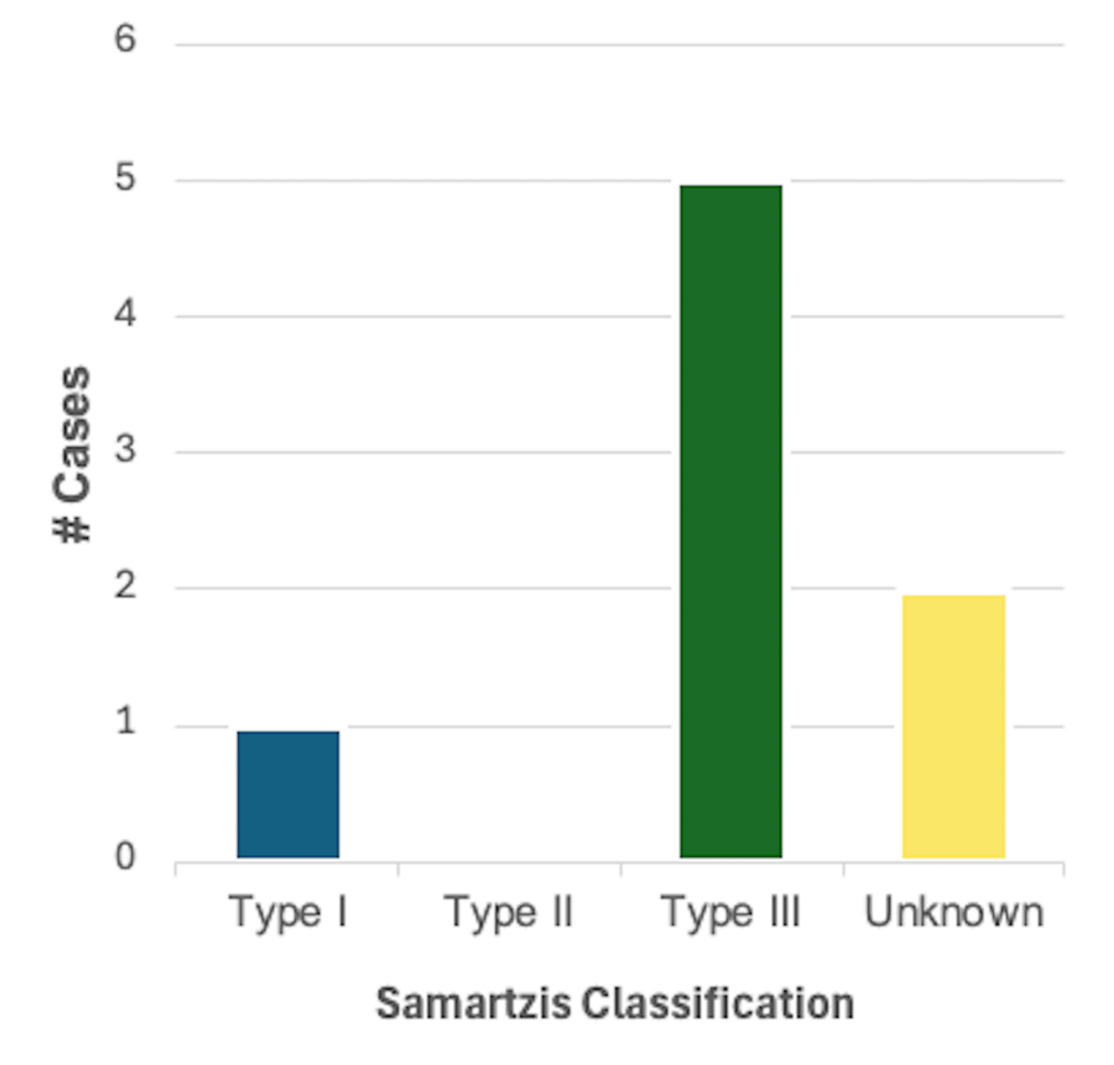
Breakdown of cases reporting one or more findings consistent with valvular disease (n = 8) based on the Samartzis classification of Klippel-Feil syndrome

Valvular disease: Valvular disease was reported in 8/46 (17.4%) cases. Of these, 2/8 (25%) were reported in male patients, 5/8 (62.5%) in female patients, and one case did not specify the biological sex of the patient. Using the Samartzis classification system, 1/8 (12.5%) cases were classified as type I KFS, 5/8 (62.5%) cases were classified as type III KFS, and two cases did not provide adequate information on the number or level of vertebral fusion. Aortic insufficiency was the most common finding in this category, reported in three cases. In addition, aortic stenosis, mitral valve prolapse, mitral valve insufficiency, tricuspid regurgitation, and fibrosis, calcification, neovascularization, and myxoid degeneration of a bicuspid aortic valve were reported in one case each. A summary of these findings can be found in Figure 5. 

Pericardial disease: Pericardial disease is a category of the ABIM Cardiovascular Disease Exam Blueprint that includes acute pericarditis, chronic pericarditis, and pericardial constriction and effusion. None of the 46 case reports analyzed in this review reported findings consistent with pericardial disease.

**Figure 6:**
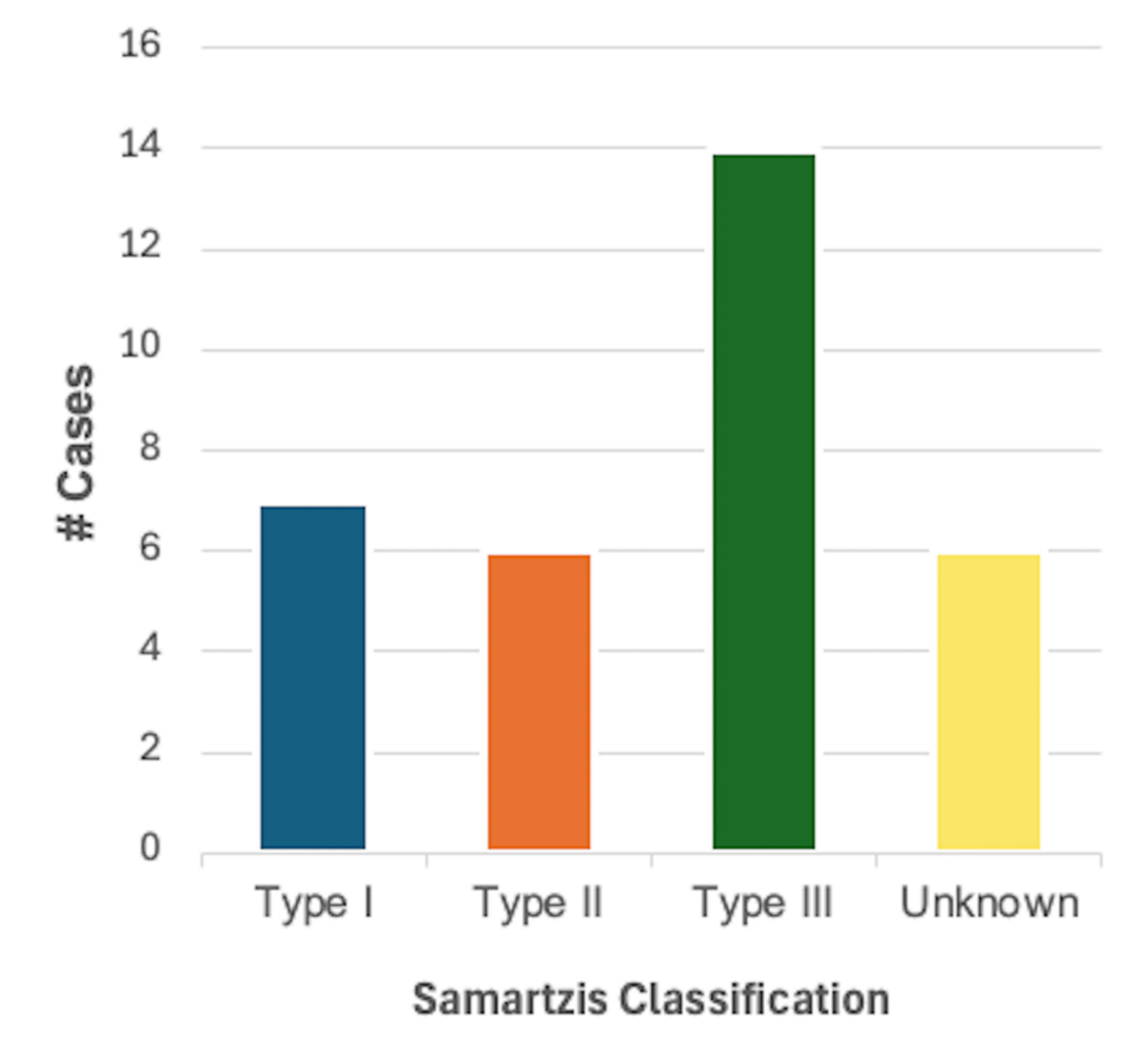
Breakdown of cases reporting one or more findings consistent with congenital heart disease (n = 33) based on the Samartzis classification of Klippel-Feil syndrome

Congenital heart disease (CHD): CHD was reported in 33/46 (71.7%) cases, making it the most common disease category. A total of 14/33 (42.4%) cases were reported in male patients, 18/33 (54.5%) were reported in female patients, and one case did not specify the biological sex of the patient. As demonstrated in Figure 6, using the Samartzis classification system, 7/33 (21.2%) were classified as type I KFS, 6/33 (18.2%) were classified as type II KFS, 14/33 (42.4%) were classified as type III KFS, and six cases did not provide adequate information on the number or level of vertebral fusion. This category of cardiovascular disease was the most diverse of those analyzed, with over 30 diagnoses described. A total of 19 cases reported a single finding within this category, while 14 cases had more than one finding consistent with CHD. Ventricular septal defect (VSD) was reported in nine cases and was the most common congenital heart defect. Bicuspid aortic valve and aberrant vertebral arteries were reported in four cases each, while dextrocardia, aberrant subclavian arteries, and coarctation of the aorta were reported in three cases each. 

**Figure 7:**
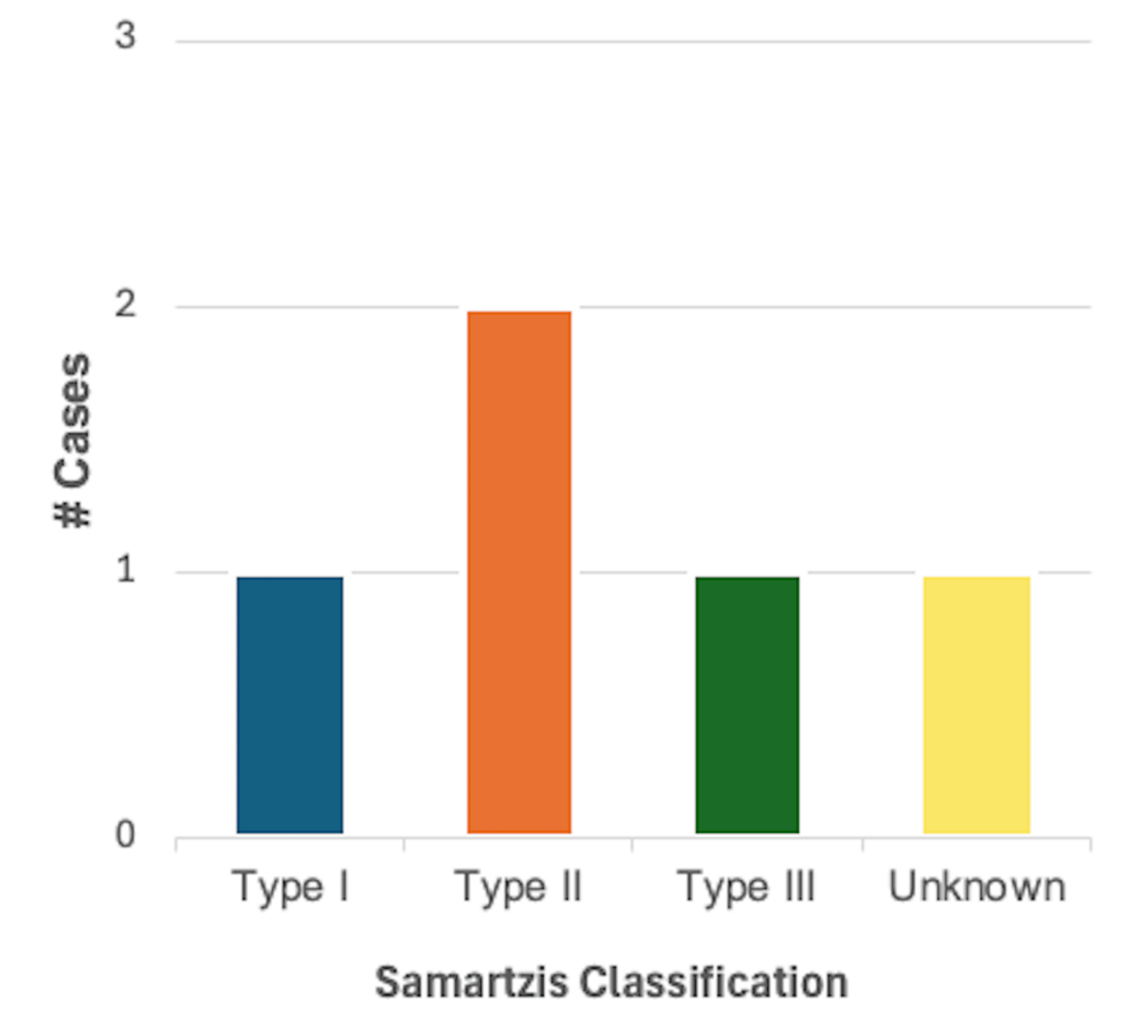
Breakdown of cases reporting one or more findings consistent with vascular disease (n = 5) based on the Samartzis classification of Klippel-Feil syndrome

Vascular diseases: Vascular disease was reported in 5/46 (10.9%) cases, of which 2/5 (40%) were in male patients and 3/5 (60%) in female patients. Using the Samartzis classification system, 1/5 (20%) cases were classified as type I KFS and type III KFS each, 2/5 (40%) cases were classified as type II KFS, and one case did not provide adequate information on the number or level of vertebral fusions. Aortic aneurysms were the most common finding in this category of cardiovascular disease, reported in three cases, while vertebral artery dissection and lateral medullary infarction were reported in one case each as well. A summary of these findings can be found in Figure 7. 

**Figure 8:**
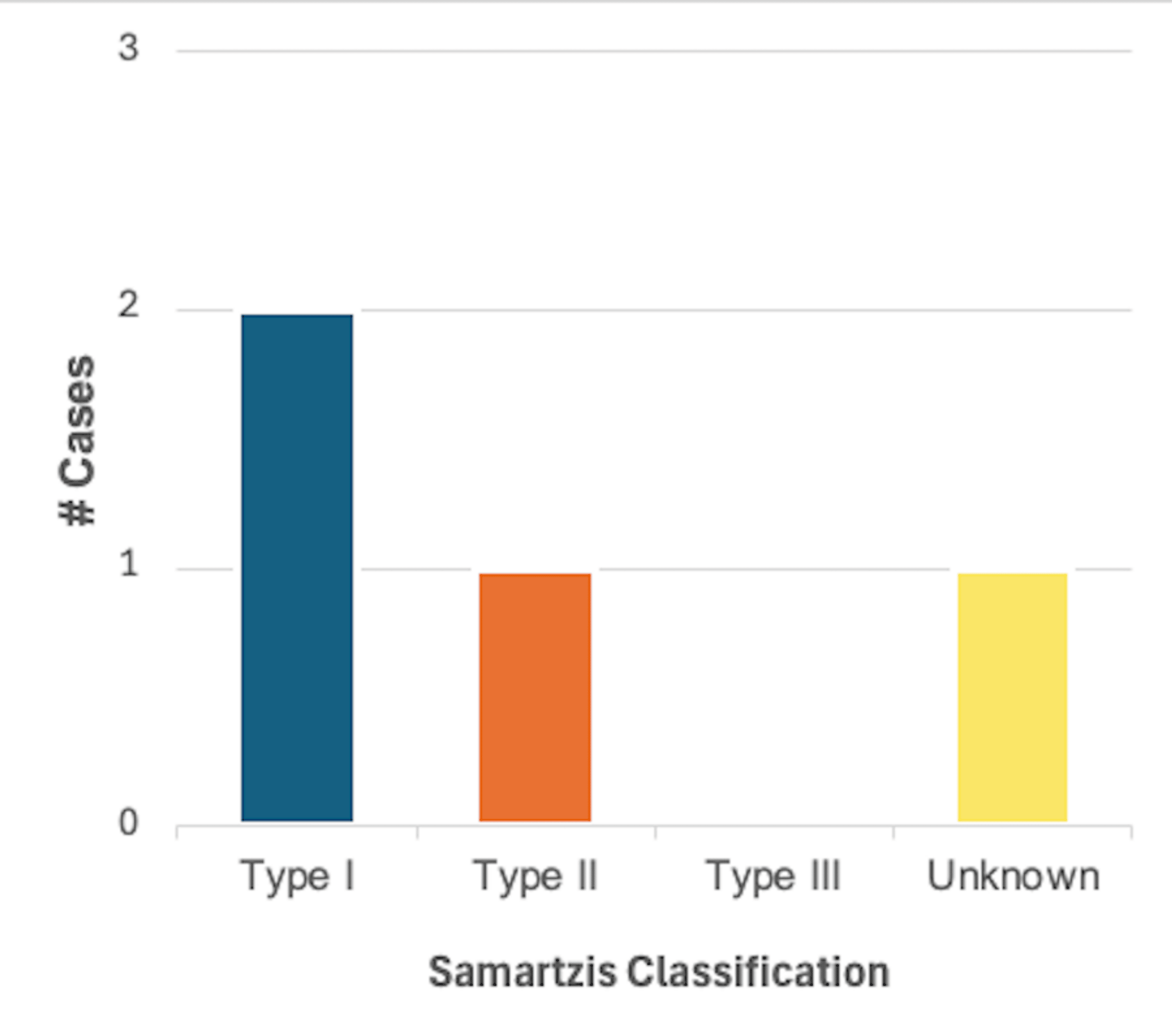
Breakdown of cases reporting one or more findings consistent with systemic hypertension or hypotension (n = 4) based on the Samartzis classification of Klippel-Feil syndrome

Systemic hypertension and hypotension: Systemic hypertension was described in 4/46 (8.7%) cases, of which 2/4 (50%) were in male patients and 2/4 (50%) in female patients. Using the Samartzis classification system, 2/4 (50%) cases were classified as type I KFS, 1/4 (40%) were classified as type II KFS, and one case did not provide adequate information on the number or level of vertebral fusions. Hypertension was the sole finding in all four cases. A summary of these findings can be found in Figure 8. 

Pulmonary circulation disorders: Pulmonary circulation disorder was reported in 1/46 (2.2%) cases. The sole case was in the form of persistent pulmonary hypertension in a 26-day-old female patient with an unspecified fusion of the cervical vertebrae. 

Systemic disorders affecting the circulatory system: A systemic disorder affecting the circulatory system was reported in 1/46 (2.2%) cases. The sole case was in the form of hypercholesterolemia in a 68-year-old male patient, which was classified as type I KFS using the Samartzis classification system. 

Other cardiovascular findings: One article included in this review described a case of a 13-year-old female patient with a medical history significant for vertebral defects, anal atresia, cardiac defects, trachea-esophageal fistula, renal anomalies, and limb abnormalities (VACTERL) association. The specific cardiac defect was not included, prompting the creation of this category. This case was unable to be categorized using the Samartzis classification system because there was not adequate information on vertebral fusion and level.

Biological Sex

**Figure 9:**
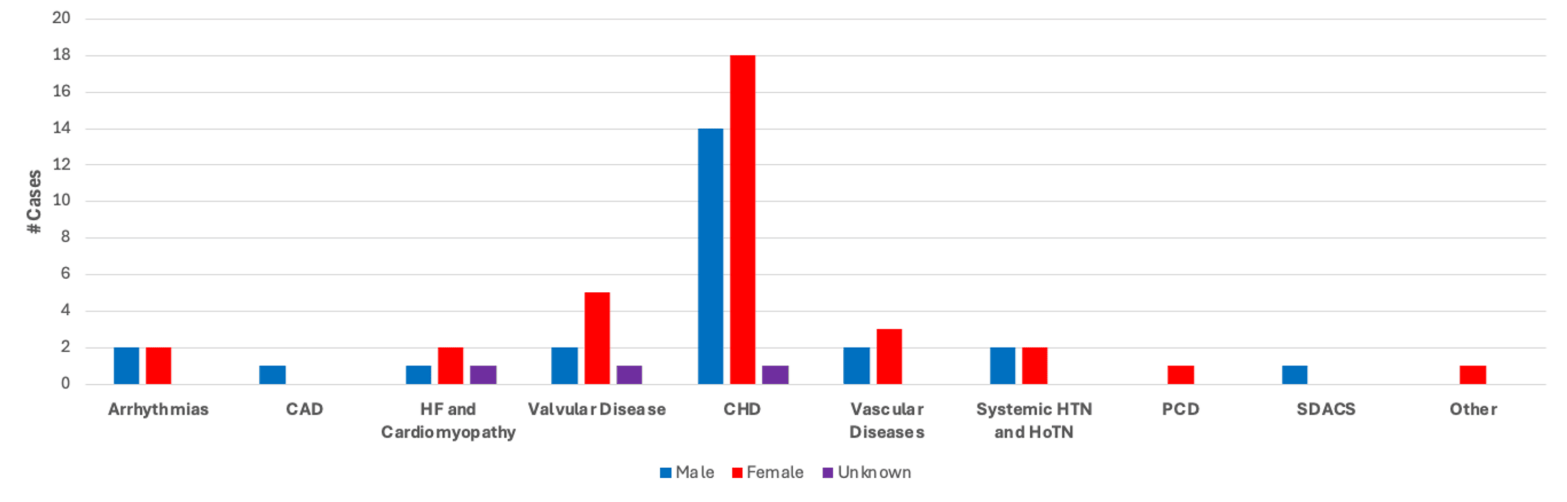
Case breakdown by cardiovascular disease category and biological sex

Of the 46 cases analyzed, 20/46 (44%) cases were reported in biological males, 25/46 (54%) in biological females, and one case did not specify the biological sex of the patient. As discussed in the previous sections, biological sex was used as a variable to determine if there was any correlation to the cardiovascular findings analyzed. Findings within the categories of arrhythmias and systemic hypertension were equally reported in males and females, while findings in the categories of heart failure and cardiomyopathy, valvular disease, CHD, and vascular disease were reported more frequently in cases with female patients than male patients. Findings within the categories of coronary artery disease, systemic disorders affecting the circulatory system, and pulmonary circulation disorders were only reported in one case each, the former two in male patients and latter in a female patient. A summary of these findings can be found in Figure 9. 

Fusion Profile

A total of 37 cases that provided adequate information on vertebral fusion level were subsequently analyzed for this section. The most common fusion profile overall was multiple fusions in the cervical region, reported in 13 cases as the sole finding and in five cases with additional fusions for a total of 18/37 (48.6%) cases. C2-C3 was the most common single vertebral fusion, reported in seven cases as the sole finding and five cases with additional vertebral fusions for a total incidence of 12/37 (32.4%) cases. A complete breakdown of the frequency of vertebral fusions among cases analyzed in this review can be found in Table 2. 

**Table 2:** Frequency of fusion profiles based on vertebral fusion level alone (n = 37) #Cases with no asterisk are those with a sole vertebral fusion specified. #Cases* are those where the specified fusion was also described with one or more other vertebral fusion

Fusion level	# Cases	Total	Frequency
AO-C1	1, 4*	5	5/37 (13.5%)
C1-C2	1	1	1/37 (2.7%)
C2-C3	7, 5*	12	12/37 (32.4%)
C3-C4	1*	1	1/37 (2.7%)
C4-C5	1, 4*	5	5/37 (13.5%)
C5-C6	1, 1*	2	2/37 (5.4%)
C6-C7	1, 1*	2	2/37 (5.4%)
C7-T1	3*	2	2/37 (5.4%)
T1-T2	2*	2	2/37 (5.4%)
T2-T3	1*	1	1/37 (2.7%)
L4-L5	1*	1	1/37 (2.7%)
Multiple cervical fusions	13, 5*	18	18/37 (48.6%)
Multiple fusions, level not specified	1	1	1/37 (2.7%)
Not specified	9	9	N/A

Fusion Profile Using the Samartzis Classification

**Figure 10:**
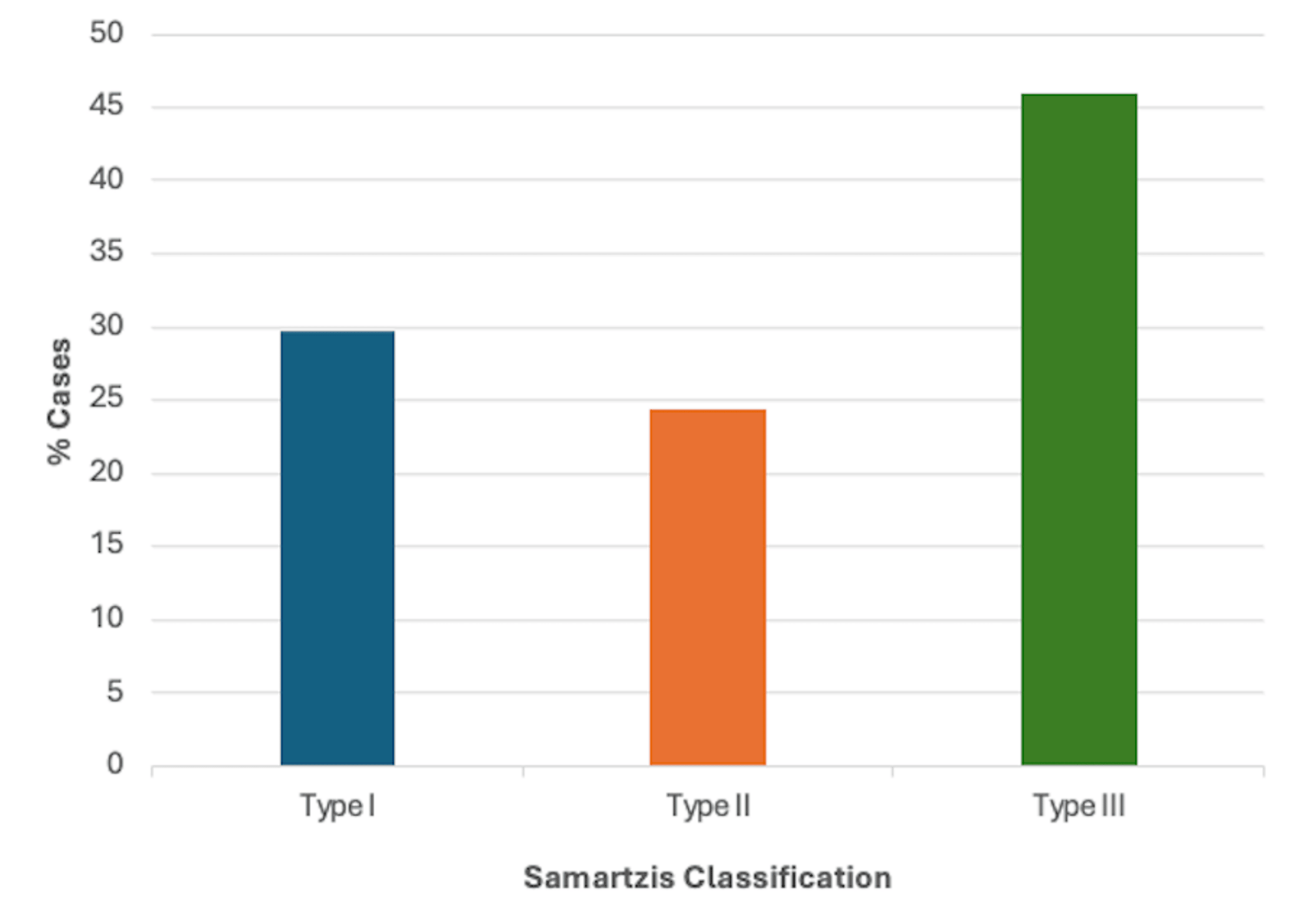
Fusion profile based on the Samartzis classification (n = 37)

As stated in the Fusion Profile section, only cases providing adequate information on vertebral fusion and level were further classified using the Samartzis classification system. A total of 17/37 cases described multiple contiguous, congenitally fused cervical segments and were therefore classified as type III KFS, making it the most common classification overall (45.9%). A total of 11/37 described a single congenitally fused cervical segment and were classified as type I KFS, while 9/37 cases described multiple, noncontiguous congenitally fused segments and were classified as type II KFS, making these the second (29.7%) and third most common classifications (24.3%), respectively. A summary of these findings can be found in Figure 10. 

Biological Sex and the Samartzis Classification

**Figure 11:**
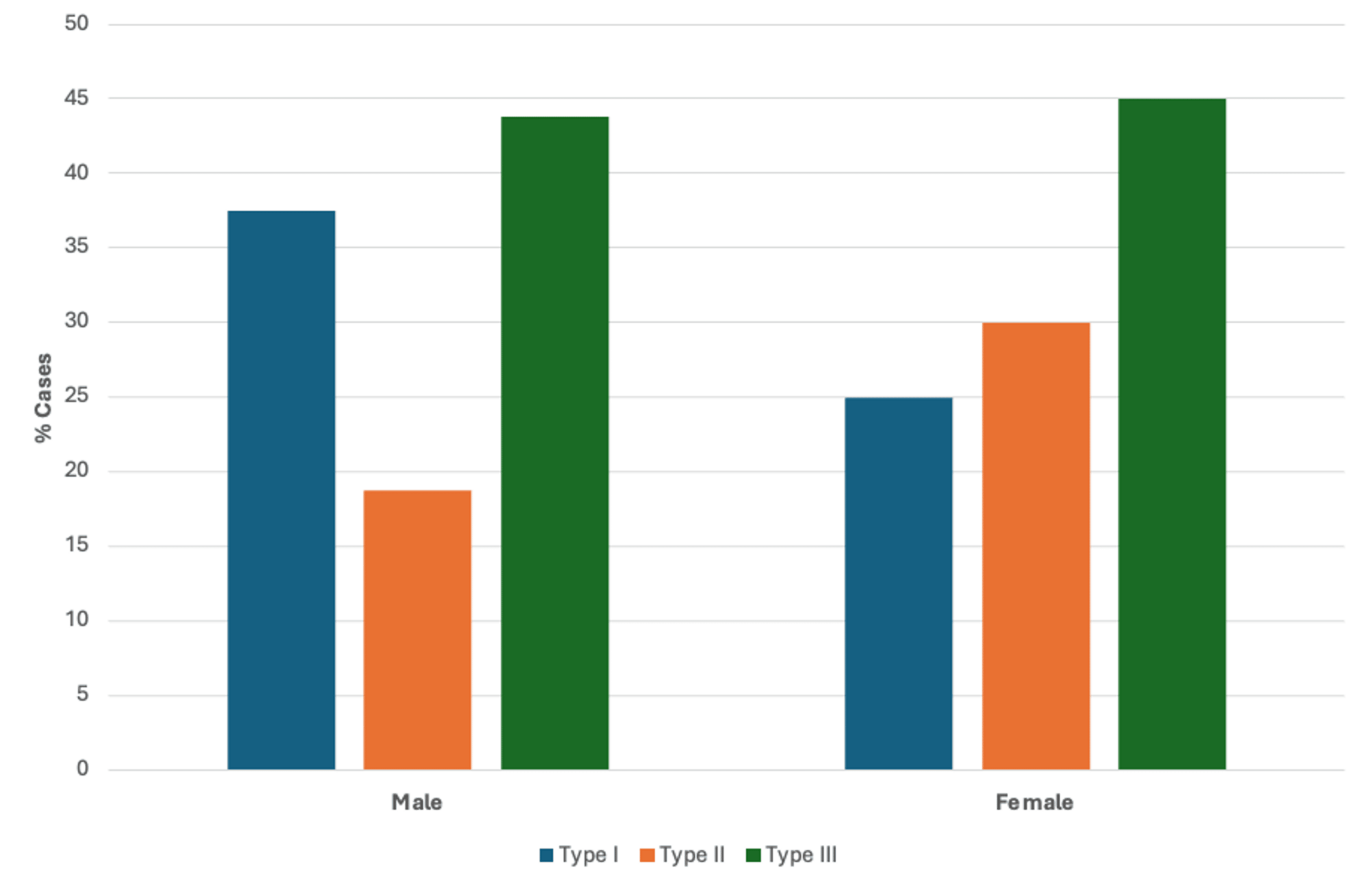
Case breakdown by Samartzis classification based on biological sex (n = 36)

Analysis was carried out to determine if a relationship existed between biological sex and the Samartzis classification of KFS. As previously mentioned, the breakdown of cases by biological sex was 20/46 (44%) males, 25/46 (54%) females, and one case did not specify the biological sex of the patient. Of these 45 cases, 36 provided information on both biological sex and vertebral fusion and were therefore grouped according to the Samartzis classification. As shown in Figure 11, type III KFS was the most common classification between both male and female patients, comprising 44% and 45% of cases, respectively. After type III, the next most common classification among male patients was type I (38%) followed by type II (19%). In cases describing female patients, after type III, type II was the next most common classification (30%), followed by type I (25%). 

Cardiovascular Findings According to the Samartzis Classification

**Figure 12:**
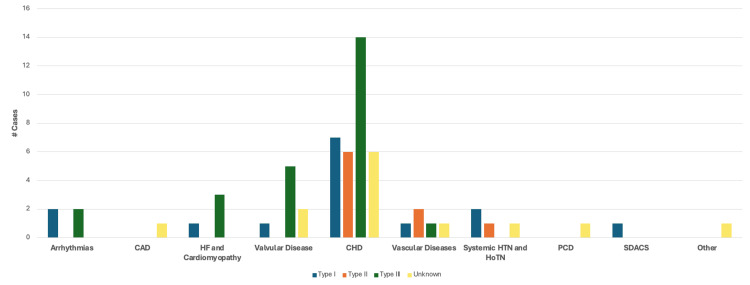
Cardiovascular findings according to the Samartzis classification CAD: Coronary artery disease; HF: heart failure; CHD: congenital heart disease; HTN: hypertension; HoTN: hypotension; PCD: pulmonary circulation disorders; SDACS: systemic disorders affecting the circulatory system

Figure 12 summarizes the number of cases describing one or more finding in each of the categories of cardiovascular disease analyzed based on Samartzis classification. Type III KFS was the most common classification reported in cases with a description of heart failure or cardiomyopathy, valvular disease, and CHD. Type II was the most common in cases describing vascular disease, and type I KFS was the most common classification reported in cases with hypertension. 

Cardiovascular Findings by Age

**Figure 13:**
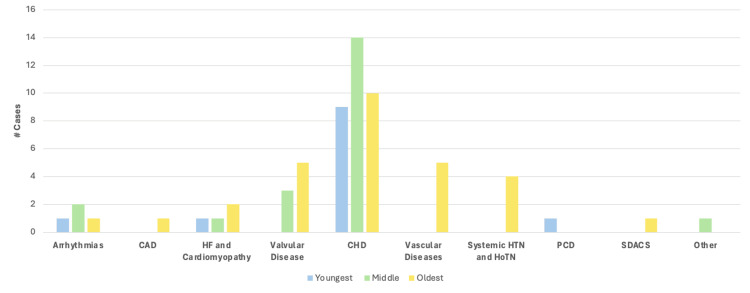
Case breakdown based on the category of cardiovascular disease based on patient age

The average age of patients included in this review was 23.3 +/- 6.8 years old (range 22 weeks’ gestation-70 years old), with a median age of 14.5 years old. Included in these calculations are two cases describing patients at 22 weeks’ gestation, whose age was set to zero for the calculation. Cases were further categorized into best fitting groups based on age, which were determined to be: in utero-36 months (youngest group), 36 months-18 years (middle group), and 18-70 years (oldest group). With this new system, the average age of each group was 5.8 months +/- 9.9 months old, 12.8 +/- 2.5 years old, and 45.3 +/- 13.8 years old, respectively. Across all ages, a description of one or more findings within the CHD category was most common, though it should be noted that the incidence decreases with age. A total of 9/10 (90%) patients in the youngest age group had one or more descriptions of CHD, 15/17 (88%) in the middle age group, and 10/19 (53%) in the oldest age group. Valvular disease was found solely in the eldest two age groups, while vascular disease and hypertension were found exclusively in the oldest group. A breakdown of the cases with findings in each category of cardiovascular diseases based on patient age can be found in Figure 13. 

Genetic Abnormalities

Of the 46 cases analyzed, eight reported a genetic abnormality. Genetic mutations were reported in MYOB18, GDF3, GDF6, and RIPPLY2. Microdeletions were reported in chromosome 19 and chromosome 12, and one case reported the patient to be HLA B27-positive. Six out of eight cases with genetic abnormalities were reported in male patients and two out of eight in female patients. All but one case included a description of CHD, including all three cases describing a mutation in GDF3 and/or GDF6. A summary of these findings can be found in Table 3. 

**Table 3:** Summary of cases reporting a genetic abnormality KFS: Klippel-Feil syndrome

Article	Age and sex	KFS classification	Genetic abnormality
Brunet et al. [16]	16-month-old M	Unknown	Homozygous nonsense variant in MYO18B
Bejiqi et al. (1) [13]	2-day-old M	Type I	Mutations in the GDF6 gene
Bejiqi et al. (3) [13]	28-month-old F	Type I	Mutations in the GDF6 and GDF3 genes
Martirosyan et al. [19]	9-year-old M	Type I	Loss of a 1-Mb region in chromosome 19 (19p13.11)
Karaca et al. [26]	13-year-old M	Type III	Apparently homozygous c.299delT: p. L100fs frameshift substitution in the RIPPLY2 gene located at 6q14.1 (recessive inheritance)
Bayram et al. [33]	21-year-old M	Type I	Mutation in GDF6 gene
Roberti et al. [34]	22-year-old F	Type II	Microdeletion of 12113.2-q13.3 (500Kb-long). Microdeletion included 26 coding genes
Cota et al. [39]	44-year-old M	Type III	HLA B27 (+)

Cardiovascular Findings in Multiple Categories

**Figure 14:**
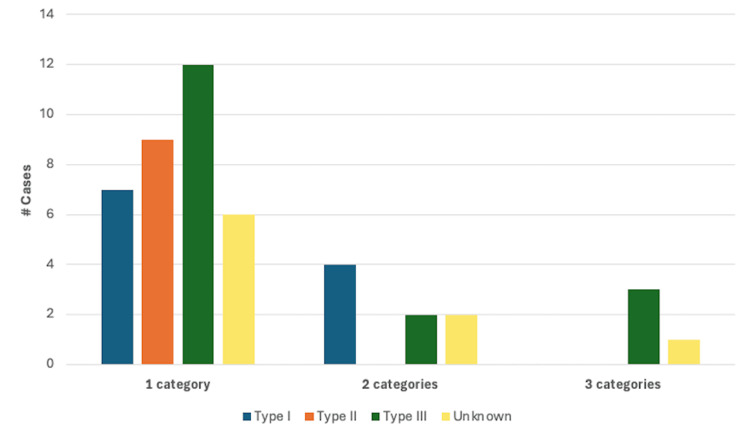
Breakdown of cases reporting one or more findings in one, two, or three categories of cardiovascular disease based on the Samartzis classification system (n = 46)

A total of 34/46 cases in this review described cardiovascular findings that could be classified in a single category of cardiovascular disease using the ABIM Cardiovascular Examination Blueprint [7]. Eight cases had findings in two categories, and four cases had findings in three categories, for a total of 12 cases reporting findings in >1 category of cardiovascular disease. Of the eight cases reporting findings across two categories of cardiovascular disease, 5/8 (62.5%) were in male patients and 3/8 (37.5%) in female patients. Using the Samartzis classification system, 4/8 (50%) were classified as type I KFS, 2/8 as type III KFS (25%), and two cases did not provide adequate information on the number or level of vertebral fusions. The most common categories with findings reported together were CHD and arrhythmia, found in 3/8 cases. Of the cases reporting findings in three categories of cardiovascular disease, 3/4 (75%) were in female patients and one case did not specify the biological sex of the patient. Using the Samartzis classification system, 3/4 (75%) cases were classified as type III KFS and one case did not provide adequate information on the number or level of vertebral fusions. Three cases reported findings in the CHD, valvular disease, and heart failure/cardiomyopathy categories, while one case reported findings in the CHD, valvular disease, and vascular disease categories. A breakdown of cases reporting one or more findings in one, two, or three categories of cardiovascular disease based on the Samartzis classification can be found in Figure 14. 

Discussion

Since its original designation in 1912, KFS has proven to be an extremely heterogenous disease with variable clinical presentations and associated conditions. Though the prevalence of KFS has historically been estimated to be between 1:40,000 and 1:42,000 live births [52], more recent studies suggest this number could be as high as 1:172 live births [53]. Along with its prevalence, the underlying cause of KFS remains unknown. Frequently cited genetic mutations in case reports include GDF3 and GDF6, MEOX-1, MYO18B, and RIPPLY2 [54-56, 17]. In a study examining a cohort of 37 Chinese patients with KFS, whole-exome sequencing with subsequent rare variant burden analysis found that BAZ1B had the highest probability of association with KFS, followed by FREM2, SUFU, VANGL1, and KMT2D [57]. Nevertheless, a definitive genetic mutation implicated in the development of KFS remains to be identified. In addition, failure of cervical somite segmentation and differentiation has been cited as contributing to the development of KFS [1], which brings into question the possibility of abnormal embryological development in the context of the cardiovascular abnormalities analyzed in this review. It is well established that the development of the primitive heart and vasculature as well as vertebral body formation and differentiation occur simultaneously and in proximity in the developing embryo [58,59]. However, the exact step or sequence of events that occurs abnormally has not been identified as it pertains to KFS but is a topic that could elucidate the initiating defect with further investigation. 

The three most commonly reported categories of cardiovascular disease found within this systematic review were CHD, valvular disease, and vascular disease. Among these, most cases were described in female patients, with a classification of type III being the most common in CHD and valvular disease and type II in vascular disease. Coronary artery disease and systemic disorders affecting the circulatory system were only reported in a single case each, both in a male patient. Otherwise, male patients did not make up a majority of cases in any of other categories of cardiovascular disease analyzed. Using the Samartzis system, after CHD, the most common category of cardiovascular disease in patients classified as type I, II, and III KFS was arrhythmia or systemic hypertension, vascular disease, and valvular disease, respectively. Finally, of the eight cases reporting findings in two categories of cardiovascular disease, the most common patient profile was type I male, while in cases reporting findings in three categories of cardiovascular disease, the most common patient profile was type III female. Overall, of the 46 cases analyzed in this review, 54% were in female patients and 46% were classified as type III KFS, making this the most common patient profile in KFS patients with some form of concurrent cardiovascular disease.

An additional benefit of conducting this review is being able to contribute data on fusion profile with and without the Samartzis classification system, as well as any associations with fusion profile, biological sex, and genetic mutations. Previous studies have demonstrated that the most common vertebral fusions seen in KFS are between C2-C3 and C5-C6 [12]. This analysis found that a fusion between C2 and C3 was indeed the most common fusion of two single vertebrae, reported in a total of 12/37 cases alone or with additional fusions. However, a fusion between C5 and C6 was only reported in 2/37 cases analyzed. The next most common vertebral fusions in this review were between AO-C1 and C4-C5, reported in 5/37 cases each, alone or with additional fusions. When cases were analyzed by biological sex and classified using the Samartzis system, the results offer a different association than has been previously published. For example, in a study looking at 22 adult KFS patients, it was reported that type I KFS was the most common, representing 55% of cases, followed by type III (27% of cases) and then type II (18% of cases) [60]. Subsequently, a study of 28 pediatric patients reported that type II KFS was the most common, representing 50% of cases, while type I and type III were equally as common and made up 25% of cases, each. In addition, after type II, this study found that female patients were more likely to be classified as type I, whereas male patients were more likely to be classified as type III [4]. The results of the present analysis differ from both studies, as type III KFS was the most common (46% of cases), followed by type I (30% of cases) and type II (24% of cases). When comparing biological sex, after type III, males were more likely to be classified as type I KFS and females were more likely to be classified as type II KFS. In terms of genetic abnormalities, eight cases described mutations, with the most common patient profile being type I male. Interestingly, of the cases that specifically reported mutations in GDF3 and/or GDF6, 2/3 were male, and all were classified as Type I KFS.

Taken together, these findings suggest that there is some association between KFS and cardiovascular disease, which is most frequently found in female patients classified as type III KFS using the Samartzis system. In addition to the thorough orthopedic, neurologic, and head-eyes-ears-nose-throat examination that has been recommended for patients with KFS [1], it would be beneficial for physicians managing KFS in patients of all ages to also include a thorough cardiovascular exam and/or provide a cardiology referral for broad screening of cardiovascular disease. Since congenital heart defects, like VSDs, increase the risk of comorbidity with a wide range of diseases [61], diagnosis at any stage could allow for better management and prevent undeterred progression of cardiovascular disease. In addition, the wide array of findings within the CHD category involving aberrant vertebral and subclavian vasculature support the prior notion that ultrasound imaging of these structures should be strongly considered in KFS patients [62,63]. Discovery of aberrant vasculature could prevent unintended damage or outcomes in the case of a procedure involving the cervical or upper thoracic region, so expansion of imaging into the upper thoracic region could be beneficial. Lastly, a baseline electrocardiogram could allow for a more thorough and longitudinal record to identify possible conduction abnormalities and contribute to an overall higher quality of care of potential cardiovascular disease in KFS patients.

Limitations

This study has potential limitations. First, the use of a single database for data collection could result in the exclusion of case reports not published in the selected database, influencing the overall case number and frequency of cardiovascular findings. However, without another piece of literature investigating the same topic, it is difficult to state the extent of this impact. Second, thorough medical histories are not reported in all case reports, so it is possible that some cases contain cardiovascular findings that were not included due to lack of reporting. Third, the rarity of KFS, combined with the inconsistency of early diagnosis, introduces the possibility of undiagnosed cardiovascular conditions in the KFS cases analyzed, especially influencing the results of the CHD category. Since the number of reports of KFS is relatively small, future studies should consider the use of multiple databases for the primary literature search. In addition, the use of the International Classification of Diseases, Tenth Revision, Clinical Modification (ICD-10) codes to categorize specific cardiovascular findings would allow for a more precise and thorough analysis.

## Conclusions

To our knowledge, this review is the first summary of the most common cardiovascular findings reported in patients diagnosed with KFS. It is established that KFS often presents with cardiovascular abnormalities, and the present analysis provides information on the specific categories of cardiovascular disease that are most common among case reports published in PubMed within the last decade. Knowledge of the cardiovascular findings commonly seen in KFS is important not only for a foundational understanding of additional disease states but also to ensure the completion of a thorough diagnostic workup. In addition to the current recommendations for the evaluation of KFS, this review provides support for the inclusion of ultrasonography in the upper thoracic region to identify aberrant vasculature off the aortic arch, a baseline electrocardiogram to ensure prompt identification of conduction abnormalities, arrhythmias, or valvular disease, and the formation of a multidisciplinary care team, including a provider specializing in cardiovascular medicine, for patients of all ages, not just pediatric patients. Further research could provide insight into whether the cardiovascular findings described here are consistent in case reports of KFS outside of those published in PubMed in the last 10 years. In addition, a more in-depth investigation of the genetic and embryologic origin of the disease could unveil the underlying mechanisms of the concurrent congenital cardiovascular defects found with KFS.
